# Expressions of Endocan in Patients with Meningiomas and Gliomas

**DOI:** 10.1155/2016/7157039

**Published:** 2016-07-27

**Authors:** Pinar Atukeren, Ahmad Kunbaz, Okan Turk, Rahsan Kemerdere, Mustafa Onur Ulu, Nursel Turkmen Inanir, Taner Tanriverdi

**Affiliations:** ^1^Department of Biochemistry, Cerrahpasa Medical Faculty, Istanbul University, Istanbul, Turkey; ^2^Department of Neurosurgery, Samatya Training and Research Hospital, Istanbul, Turkey; ^3^Department of Neurosurgery, Cerrahpasa Medical Faculty, Istanbul University, Fatih, 34098 Istanbul, Turkey; ^4^Department of Forensic Medicine, Uludag University, Bursa, Turkey

## Abstract

*Objective*. Endocan has been shown to be a marker for several cancers and may show degree of malignancy. The aim of this study is to assess tissue levels of endocan in common brain tumors, namely, meningiomas, low-grade gliomas (LGGs), and high-grade gliomas (HGGs).* Patients and Methods*. Endocan was assayed by commercially available enzyme linked immunosorbent assay (ELISA) kits in a total of 50 brain tumors (20 meningiomas, 19 LGGs, and 20 HGGs) and 15 controls. The results were compared to control brain tissues.* Results*. Each tumor group showed significant higher levels of endocan compared to controls (*p* < 0.05). In addition, endocan levels showed steady increase from the least (meningiomas) to the most (HGGs) malignant tumors and positive correlation was noted between the degree of malignancy and endocan level (*p* = 0.0001).* Conclusion*. Endocan, a vital molecule for angiogenesis, is expressed in common brain tumors and results suggest that endocan could be a marker for malignancy.

## 1. Introduction

Gliomas are the commonest intra-axial brain tumors generally divided into two groups for practical purposes: low-grade (grades I and II) and high-grade (grades III and IV) gliomas [1]. High-grade gliomas (HGGs) are the most feared brain tumors since they have a high recurrence rate, especially in case of glioblastoma multiforme (grade IV); the median survival time is almost 15 months despite the current modern treatment modalities. Low-grade gliomas (LGGs) are also common and they can upgrade within 5 to 7 years and finally behave as HGGs.

On the other hand, the most common extra-axial tumors in the intracranial compartment are meningiomas, originated from the cap cells of arachnoid. These tumors were classified by the World Health Organization into three grades (grades from I through III); grade III is the malignant form and recurrence is very high [1].

Since we do not have still curative treatment with respect to many solid cancers, identifying a specific marker or a target becomes vital or understanding the molecular basis behind these tumors may lead us to developed curative treatment. The rapidly expanding data on endothelial cell-specific molecule-1 (ESM-1) or endocan shows that it is a soluble dermatan sulfate proteoglycan (PG), freely circulating molecule in the blood, and present primarily on the cell surface, in the extracellular matrix and body fluids [2]. Recent studies suggest that this molecule may be used as a target in the treatment of several cancers or may be a marker for the some tumors [3–6]. Endocan is produced by endothelial cells and includes a protein core, dermatan sulfate [7, 8]. Its overexpression in several disorders, such as sepsis [9], cancers [3–6], or inflammatory conditions, suggests that these molecules may be involved in the pathogenesis. The large body of evidence currently indicates that endocan not only is a biomarker of neoangiogenesis but also shows tumor progression when expressed by tumor itself. This molecule has been studied in serum or tumor tissues or tumor lines of several other tumors but there is little information about the levels of endocan in the brain tumors and the present study is the first prospective study to show expressions of endocan in patients with common brain tumors, namely, gliomas and meningiomas. A few studies related to endocan have been evaluated in pituitary adenomas [6, 10] and only one study showed expressions of this molecule at both the mRNA and protein levels in human glioma cell lines and used tissue sections from gliomas to determine localization of endocan immunoreactivity in situ [5].

## 2. Patients and Methods

### 2.1. Study Population

This study was conducted in Istanbul University, Cerrahpasa Medical Faculty and Departments of Neurosurgery and Biochemistry, during the year 2015. All patients or next of kin were fully informed and ethical approval for this study was obtained from the Human Investigations Committee at Istanbul University. We excluded the patients who had any kind of chronic or acute infection, immunological and metabolic diseases, neoplastic disease of other organ systems, any cardiovascular diseases, and recent major surgical procedure at the time of tumoral tissue collection, in which endocan status might be affected.

### 2.2. Patients and Controls

A total of 59 adult patients and 15 controls served as subjects in this study. The tumor groups were divided into three groups:* meningiomas* were diagnosed in 20 patients and the majority were grade I (17 patients). In* LGGs*, 19 patients were included and the tumors were diagnosed with grade I in one patient and grade II in 18 patients. The last group of tumor was composed of 20 patients with HGGs: grade III in 7 and grade IV (glioblastoma multiforme-GBM) in 13 patients. Control group consisted of 15 adult victims who died as a result of traffic accident or fall from height. All had undergone autopsy procedure in the Department of Forensic Medicine in Uludag University, Bursa, Turkey, and Morgue Department, Council of Forensic Medicine, Bursa, Turkey. No subject showed gross pathology in the brain during the autopsy procedures.

### 2.3. Specimen Handling

A total of 74 tissue samples were assayed for endocan. For each patient, tumor tissues were collected during surgery, and from the control group, brain tissues were obtained during the autopsy procedure. Brain tissues from the controls were obtained within the first 4 h following death. As soon as possible, each sample was stored at −80°C until being assayed.

### 2.4. Preparation of Tissue Samples

Brain tumor and cadaver brain tissue samples were washed in cooled 0.9% NaCl and placed on an ice-cold plate, incised, and weighed. The samples were then immediately frozen in liquid nitrogen until they were homogenized. Tissue samples were homogenized manually in homogenizing buffer (100 mM KH_2_PO_4_–K_2_HPO_4_), to obtain 20% homogenates, with a tissue grinder fitted with a Teflon pestle for the measurement of adhesion molecules' levels and for total protein determination. The homogenates were sonicated with MSE sonicator two times at 30 sec intervals on ice, with a power output of 38 watts. Supernatant fractions were obtained by the centrifugation of the homogenates in 15000 ×g for 15 min. During aliquot preparation, supernatant fractions were maintained at +4°C in dim light. The supernatant fractions were divided into aliquots (one for each assay) and immediately stored at −80°C (for 2 weeks maximum) for the measurement of biochemical parameters.

### 2.5. Assays of Endocan

Endocan levels were measured with commercially available enzyme linked immunosorbent assay (ELISA) kits (YH Biosearch Laboratory, Shanghai, China) based on biotin double antibody sandwich technology. Actual levels of this parameter in the samples were determined from the standard curves.

### 2.6. Measurement of Total Protein Content

Total protein content of the samples was measured by using the modified method of biuret with some volumetric modifications, proposed by Itzhaki and Gill [11]. Principle of the method is based on formation of a Cu^2+^-protein complex production, a violet-colored chelate product, which can be measured by absorption spectroscopy at 540 nm. Biuret reagent was prepared by adding 3 g of CuSO_4_·5H_2_O and 9 g of sodium potassium citrate to 500 mL of 0.2 NaOH, followed by the addition of 5 g of KI. Biuret reagent was added to all samples and standards at a volume of 1 : 20. After a 20-minute incubation period, colorimetric reading was performed for all specimens. Recorded absorptions of the samples at 540 nm were compared with the protein standards.

### 2.7. Statistical Analysis

We used a commercially available statistical software package (SPSS version 14.0 Inc., Chicago, IL, USA) for all the statistical analyses. The mean ± standard deviations (±SD) were calculated for each parameter. For all comparisons, the nonparametric Mann-Whitney *U* test was used as a statistical method. Nonparametric Spearman's correlation test was used for correlations. Differences were considered statistically significant if the probability value was less than 0.05.

## 3. Results

**Figure 1:**
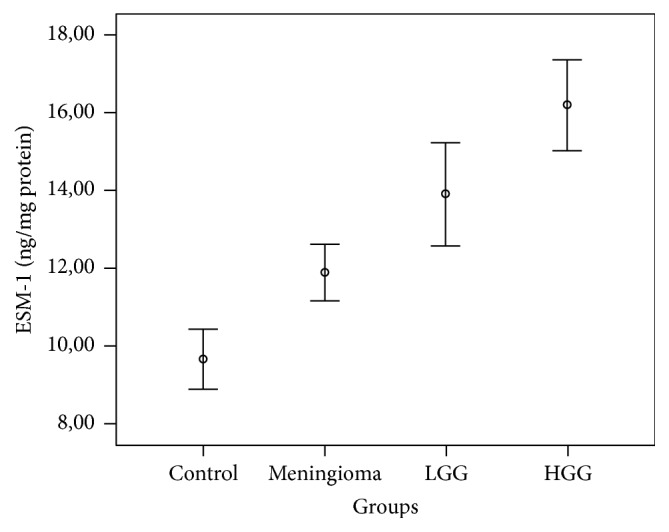
*Graph *showing a comparison of levels of endothelial cell-specific molecule-1 (ESM-1) in the groups and the differences between all four groups were significant (*p* < 0.05).* Circles *represent the means ± standard errors of the means and* bars *denote the range of values.

Statistical results including the mean (±SD) and probability (*p*) values are summarized in Tables 1 and 2 and Figure 1.

### 3.1. Endocan in the Groups

Mean level of endocan showed a steady incline from the controls to the most malignant form of the tumor groups. All the tumor groups showed significant higher levels compared to the controls (*p* < 0.05) and the highest mean level was found in HGG group. Considering each tumor group, again lowest mean level was noted in meningioma group (most benign) but highest levels again were found in HGG (most malignant) group. The level with respect to LGGs was in between. Comparisons between the tumor groups also displayed significant differences. The most significant difference was expectedly noted between meningiomas and HGGs (*p* = 0.0001). Correlation analysis showed that endocan level positively correlated with the degree of malignancy (*r* ^2^ = 0.36, *p* = 0.0001); as the degree of malignancy increases, endocan level increases.

#### 3.1.1. Correlations

While this paper was being written, mean follow-up times for meningiomas, LGGs, and HGGs were found to be 27.2 ± 12.5, 24.1 ± 10.8, and 21.5 ± 13.2 months, respectively. At the last visit, all twenty patients with meningiomas were alive and head magnetic resonance imaging (MRI) showed no recurrence. Three patients diagnosed with grade II meningioma (atypical) received radiotherapy and only one patient in the group had seizure which was under the control by one antiepileptic medication.

There was no death in LGGs group and 3 showed unremarkable progression of the residue which was decided to be followed up radiologically in 3 months' interval. No patient in this group received radio- or chemotherapy. Four patients showed seizure which was present since surgery and they were on antiepileptic medication.

As expected, the most dramatic changes were noted in HGGs group. All received radio- and chemotherapy. Eleven (84.6%) of the thirteen patients with grade IV glial tumor died during the follow-up period. The two patients who are still alive showed no radiological recurrence and are free of clinical symptoms. Seven patients diagnosed with grade III glial tumors are still under our follow-up and all patients are alive and are still on antiepileptic medication.  Table 2 shows endocan levels and follow-up times for each patient.

Correlation analysis between endocan levels and overall survival within the tumor groups showed negative relationship (*r* ^2^ = −0.03, *p* = 0.01). The higher the endocan levels, the shorter the survival time.

## 4. Discussion

It has been demonstrated that endocan is expressed from endothelium of vascularized organs but surprisingly highly vascularized organs such as heart, pancreas, liver, and brain do not contain endocan [2, 5, 7]. This finding suggests that this molecule is expressed upon activation rather than resting state of the cell. Endocan has diverse functions in both normal and pathological conditions. Expression of endocan was found to be increased in sepsis [9], inflammation [8], and cancers [2–6, 10, 12]. It suggests that this molecule is involved in angiogenesis during the development of a tumor. We know very well that angiogenesis or formation of new vessels is a sine qua non or key event in tumor progression. Angiogenesis-related factors or proangiogenic molecules are upregulated together with endocan which promotes mitogenic and migratory activities of vascular endothelial growth factors A and C which are strong angiogenic molecules (reviewed in [12]). Lung, colon, liver, kidney, stomach, prostate, and bladder tumors showed high expressions of endocan in the newly formed vessels [2–6, 8, 13, 14]. Studies including brain tumors are very limited and a few studies showed endocan may be used as a marker for invasiveness in pituitary adenomas [6, 10]. Expressions in endothelial cells of vessels and tumor tissue itself suggest that endocan is an angiogenesis marker and may be an essential molecule in tumor formation and growth and associated with aggressive behavior. In one study, endocan expression was always found on the endothelial cells of newly formed vessels and tumor cells of GBM, which is the most aggressive tumor of the brain and characterized by extensive angiogenesis and proliferating multilayered capillary vessels [5]. Interestingly, they found no endocan in LGGs and normal cerebral tissues far from the tumor [5]. Strong association between endocan expression by endothelium and recurrence, invasiveness, and poor prognosis has been demonstrated in hepatocarcinoma [4], pituitary adenoma [6, 10], and GBM [5]. More importantly, serum levels of endocan were associated with early detection of colorectal cancer and liver carcinoma [15, 16]. These findings underline that endocan may function in angiogenesis-driven tumorigenesis in which angiogenesis-directed therapies could help in the control of these tumors. Depending on the current literature, it is obvious that endocan is highly restricted to endothelial cells, especially vascular endothelial cells, and it can be used as a new endothelial cell activation marker. Thus, endocan may be a marker of malignancy or aggressive behavior since expression in tumor areas is closely associated with hypoxia which is a strong stimulator of angiogenesis.

The current study is the first prospective study to show expression of endocan in common brain tumors, namely, gliomas and meningiomas. No study noted so far showed endocan expressions in meningioma. Previous studies showed that endocan expression is not noted in normal brain tissue although it is one of the highly vascularized organs [2, 5, 7]. We used control cerebral tissues from adults who underwent autopsy procedure because of death due to fall or traffic accidents. We were very careful to get the tissues from the brains which were not affected directly from the trauma. We identified endocan expression from the cerebral cortices of the controls. This finding seems to be opposite to the previous study in which they did not find endocan in normal cerebral tissue [5]. This difference may be due to the different method that we used ELISA which measures cytoplasmic endocan in contrast to the one single study [5] that used immunohistochemistry in gliomas, which measures nuclear endocan. By depending solely on our findings, we cannot speculate that normal brain tissues also contain endocan; however, we can underline that endocan expression may be induced by hypoxia in the control brains because we collected the normal cerebral tissues within the first 4 hours of the trauma. Our findings related to the tumors are very well in line with the current literature, especially with the study which is the only single study including gliomas [5]. The mean levels in the tumors showed significantly higher levels compared to the controls. Considering the three tumor groups, the highest level was found in HGGs, followed by LGGs and meningiomas. Comparisons between the tumor groups also demonstrated significant differences and positive correlation between the degree of malignancy and endocan levels was noted; the most aggressive the tumor, the higher the level. Furthermore, endocan levels correlated with overall survival but we have to underline that our results should be taken into consideration carefully because of less number of patients included here. In contrast to the previous study [5], we found endocan expressions in LGGs too. Meningiomas are highly vascularized tumors which are generally homogeneously contrasted on the magnetic resonance imaging and show dense vascular supply on angiography. Thus, endocan expression higher than control is expected in meningiomas since dense vascular supply is one of the hallmarks in meningiomas. Finding of endocan expressions in LGGs in the current study suggests that any grade of gliomas is also angiogenesis-driven and for the tumor progression and/or upgrading of a LGG, newly vessels continuously are formed and endocan can be expressed on the endothelium of newly formed vessels. Furthermore, the mean level of endocan expression was found to be higher compared to meningioma and may reflect higher level of microvascular remodeling and growth even in LGGs.

## Figures and Tables

**Table 1:** The mean (±SD) value and results of statistical comparisons of endothelial cell-specific molecule-1 between the groups.

Parameters/groups	Controls	Meningioma	LGG	HGG	*p* value
ESM-1 (ng/mg protein)	9.66 ± 1.45	11.8 ± 1.62	13.9 ± 2.90	16.1 ± 2.59	

*Comparisons*					
Controls versus meningioma					0.001^*∗*^
Controls versus low-grade gliomas					0.0001^*∗*^
Controls versus high-grade gliomas					0.0001^*∗*^
Meningioma versus low-grade gliomas					0.01^*∗*^
Meningioma versus high-grade gliomas					0.0001^*∗*^
Low-grade gliomas versus high-grade gliomas					0.02^*∗*^

C: controls; ESM-1: endothelial cell-specific molecule-1; HGG: high-grade glioma; LGG: low-grade glioma; M: meningioma.

^**∗**^ *p* < 0.05.

**Table 2:** Overall survival and endocan levels (ng/mg protein) in patients studied here.

Number	Endocan levels in the groups				Follow-up (months) MGs/*LGGs*/HGGs
	Controls	MGs	LGGs	HGGs	
1	8.12	12.27	14.55	15.62	36/*24*/9^*∗*^ (D)
2	9.76	14.50	12.65	19.05	36/*48*/36
3	11.08	12.98	13.20	22.30	12/*36*/60
4	10.91	11.62	18.90	17.50	36/*12*/12^*∗*^ (D)
5	10.72	12.02	11.77	14.36	16/*48*/15^*∗*^ (D)
6	7.92	10.91	12.27	12.78	60/*13*/36
7	8.41	9.23	10.78	18.90	24/*24*/12^*∗*^ (D)
8	8.77	11.77	12.98	14.55	36/*14*/15^*∗*^ (D)
9	11.20	10.72	9.25	16.71	36/*15*/18^*∗*^ (D)
10	10.32	9.21	17.62	17.62	36/*18*/12^*∗*^ (D)
11	8.41	11.21	10.91	15.02	36/*24*/30^*∗*^ (D)
12	7.65	12.36	9.23	16.71	23/*24*/30
13	12.04	14.32	14.52	12.65	13/*24*/42
14	8.62	10.27	14.60	10.91	23/*16*/18
15	11.08	11.41	17.30	18.09	12/*36*/12^*∗*^ (D)
16	—	14.55	14.50	17.52	13/*24*/13^*∗*^ (D)
17	—	14.36	18.62	15.94	24/*19*/12^*∗*^ (D)
18	—	12.50	16.02	15.94	36/*26*/15^*∗*^
19	—	10.91	14.50	14.20	12/*13*/16^*∗*^
20	—	10.65	—	17.60	24/—/18

D: died; HGGs: high-grade gliomas; LGGs: low-grade gliomas; MGs: meningiomas.

**∗**denotes grade-IV astrocytoma (glioblastoma multiforme).

## Additional Points

The authors want to acknowledge some limitations in this study. The first limitation is that we included small sample size of the patients in each group. This is because we included consecutive patients who gave permission to us and because of other strict inclusion criteria. We especially wanted to see whether endocan levels increased on common brain tissues and especially in meningiomas and LGGs in which results are preliminary. Thus, in spite of small sample size, this study may give an idea to the neurosurgeons who want to develop further studies which should include larger sample size of patients. Second limitation is that we did not perform immunohistochemistry studies on tumor tissues which would be more validated and further studies on brain tumors should be designed to include immunohistochemistry and the third limitation in this study is that serum levels were not studied. Our next study will include LGGs in which we will measure serum levels of endocan before and after surgery to see whether there is any correlation between serum levels of endocan and surgical removal. In conclusion, depending on the current literature and our own findings, endocan may be a marker of tumor aggressiveness in common brain tumors: as the grade of the tumor increases, endocan level increases. Since studies including brain tumors are very limited, further studies are needed to verify our findings and future results may lead us to develop monoclonal antibody directed to endocan to prevent aggressiveness of the brain tumors.
